# Vitamin E administration as preventive measures for peritoneal/intra-abdominal adhesions: A systematic review and meta-analysis

**DOI:** 10.1016/j.amsu.2022.104225

**Published:** 2022-07-31

**Authors:** Taufik Sudirman, Mochammad Hatta, Prihantono Prihantono, Agussalim Bukhari, Tjahyadi Robert Tedjasaputra, Hendry Lie

**Affiliations:** a Department of Surgery, Faculty of Medicine, Pelita Harapan University, Tangerang, Indonesia; b Department of Molecular Biology and Immunology for Infectious Disease, Faculty of Medicine, Universitas Hasanuddin, Makassar, Indonesia; c Department of Surgery, Faculty of Medicine, Universitas Hasanuddin, Makassar, Indonesia; d Department of Nutritional Sciences, Faculty of Medicine, Universitas Hasanuddin, Indonesia; e Division of Gastroenterology, Department of Internal Medicine, Siloam Hospital Lippo Village, Tangerang, Indonesia

**Keywords:** Peritoneal, Abdominal, Adhesions, Vitamin E, Prevention

## Abstract

**Background:**

Peritoneal adhesion still becoming a common complication after abdominal surgeries and become a significant threat to digestive surgeons nowadays. Vitamin E might offer benefits for preventing peritoneal adhesions because of its antioxidant, anti-inflammatory, and anti-fibroblastic properties. This study sought to analyze the relationship between vitamin E administration and peritoneal/intra-abdominal adhesions in rat models.

**Methods:**

Potential articles were searched by using specific keywords on Scopus, PubMed, PMC, and Cochrane Library databases until March 12th, 2022. All published studies on vitamin E and peritoneal/abdominal adhesions in rat models were collected. Statistical analysis was performed by using Review Manager 5.4 software.

**Results:**

A total of 9 studies were included in the final analysis. Pooled analysis of the evidences yielded an association between vitamin E and decreased incidence of substantial peritoneal/intra-abdominal adhesions (RR 0.46; 95%CI: 0.33–0.64, *p* < 0.00001, *I*^*2*^ = 61%, random-effect modeling); and reduction in the mean grade of adhesions (Mean Difference −1.53; 95%CI: −2.00, −1.06, *p* < 0.00001, *I*^*2*^ = 98%, random-effect modeling).

**Conclusions:**

This study proposes that vitamin E supplementation might offer benefits in the prevention of peritoneal/intra-abdominal adhesions. More in-vivo studies with larger sample sizes and proper methods are still needed to confirm the results of our study. If possible, studies on humans might also be warranted.

## Highlights

• Peritoneal adhesion still become common problem after abdominal surgery.
• Our study indicates vitamin E can reduce incidence of peritoneal adhesions.
• Vitamin E can also reduce the mean grade of abdominal adhesions.

## 1 Introduction

Peritoneal adhesions remain a common complication following abdominal surgery with the incidence ranging from 93 to 100% after upper abdominal laparotomies and 67–93% after lower abdominal laparotomies [1,2]. Nearly 15–18% of these adhesions require surgical re-intervention. Of all small bowel obstructions, 65–75% of those cases are caused by peritoneal adhesions, meanwhile, 32% of acute intestinal obstructions are also caused by peritoneal adhesions [1,2]. Some factors which can cause peritoneal adhesions are intra-abdominal infections, surgical trauma, ischemia, heat injury, and host reaction to a foreign body [3]. All of these data showed that peritoneal adhesions still become a serious problem for digestive surgeons.

Several interventions have been tried to prevent the occurrence of peritoneal adhesions. Hyaluronic acid applied to a layer of carboxymethylcellulose is one of the agents which have been studied to reduce the incidence of peritoneal adhesions. However, this agent may cause a significant increase in the anastomotic leakage incidence when the membrane is applied in direct contact with the anastomosis [4].

Another agent which is currently studied for peritoneal adhesions is vitamin E. Vitamin E is one of the vitamins which can dissolve in lipids and can be found in all cell membranes [5]. Vitamin E can serve as an antioxidant, anti-inflammatory, anti-coagulant, and also anti-fibroblastic [5]. With those potentials, vitamin E might offer beneficial effects toward the prevention of adhesion formation. However, the evidence regarding the efficacy of vitamin E administration in preventing peritoneal or intra-abdominal adhesions is still unclear. This systematic review and meta-analysis aim to summarize the evidence regarding the benefit of vitamin E administration in reducing the peritoneal/intra-abdominal adhesions in rats models which may also be useful to guide further experiments in humans.

## 2 Materials and methods

### 2.1 Eligibility criteria

We conducted a systematic review and meta-analysis study from animal experimental studies. We registered this study protocol in PROSPERO (CRD42020181430). Append research in this systematic review and meta-analysis were chosen as most likely attaining the coming criteria: follow the PICOS framework (P: Populations – rats/mice from any types or species which undergone peritoneal or intra-abdominal adhesion induction procedure through any available methods; I: Interventions – vitamin E administration through any methods with any dose; C: Comparator/Control – did not receive vitamin E or only receiving placebo; O: Outcomes – incidence of substantial peritoneal or intra-abdominal adhesions and mean grade of adhesions; S: Study Design – animal experimental studies). All studies besides original articles (letter to editor, correspondence, or review articles), any studies in humans (clinical trials, cohort, case-control, case-series, case report), studies reported other than in English language, research focusing on animals other than rats/mice were excluded.

### 2.2 Search strategy and study selection

Searching of the literature was done systemically towards articles with English-language restrictions which were sourced from four databases (PubMed, PubMed Central/PMC, Scopus, and Cochrane Library). Combined keywords such as "vitamin E" OR “alpha-tocopherol” AND “peritoneal adhesion” OR "abdominal adhesion" were used to filter the intended articles from the period 1950 until March 12th, 2022. The details of this study's searching strategies can be seen in Supplementary Table 1. The first step involves finding eligible studies through titles and abstract screening. Additional evaluation of references from found eligible studies was also conducted to search for more potential articles. Preferred Reporting Items for Systematic Reviews and Meta-Analyses (PRISMA) diagram shows the strategy we employed during our study [6]. We also have fully complied with the AMSTAR 2 criteria [7].

### 2.3 Data extraction and quality assessment

Two authors conducted the data extraction. Extraction form was developed to list information about the study such as its sample characteristic, adhesion induction procedure, vitamin E dosage, number of rats/mice treated with vitamin E, the control group in included studies, as well as the outcome of peritoneal or intra-abdominal adhesions.

We focused the outcomes of our study on substantial peritoneal or intra-abdominal adhesions and the mean grade of adhesions. Intraperitoneal or intra-abdominal adhesions were graded according to the classification reported by Nair et al. [8], which consist of the followings: (1) grade 0: Complete absence of adhesions; (2) grade 1: Single band of adhesions, between viscera, or from one viscus to abdominal wall; (3) grade 2: Two bands: between viscera or from viscera to abdominal wall; (4) grade 3: More than two bands: between viscera, or viscera to the abdominal wall, or whole of intestines forming a mass without being adherent to abdominal wall; (5) grade 4: Viscera directly adherent to the abdominal wall, irrespective of number and extent of adhesive bands. Grade 0 and grade 1 were considered insubstantial adhesions, while grade 2 until grade 4 were further classified as substantial adhesions.

Two authors assessed the quality of each study involved in this review independently. SYRCLE's risk of bias (RoB) tool was used to evaluate the quality of animal experimental studies [9]. The assessment process included reviewing the 10 different entries which are related to 6 types of bias: selection bias, performance bias, detection bias, attrition bias, reporting bias, and other biases, then each entry and study was assigned to low (+), high (−), or unclear (?) risk of bias.

### 2.4 Statistical analysis

Meta-analysis was done using Review Manager 5.4 (Cochrane Collaboration). Application of the Mantel-Haenszel formula with random-effect models regardless of heterogeneity was employed to calculate the risk ratio (RR) and its 95% confidence interval (95% CI) for the incidence of substantial adhesions outcome. Meanwhile, we use the Inverse-Variance formula with random-effect models to calculate the mean difference (MD) and its standard deviations (SD) for the mean grade of adhesions outcome. In this meta-analysis, heterogeneity between studies was assessed by the I-squared (I^2^; Inconsistency). The I^2^ statistic with a value of <25%, 26–50%, and >50% were considered as low, moderate, and a high degree of heterogeneity, respectively. Funnel plot analysis was utilized to assess the publication bias risk qualitatively.

## 3 Results

### 3.1 Study selection and characteristics

**Fig. 1:**
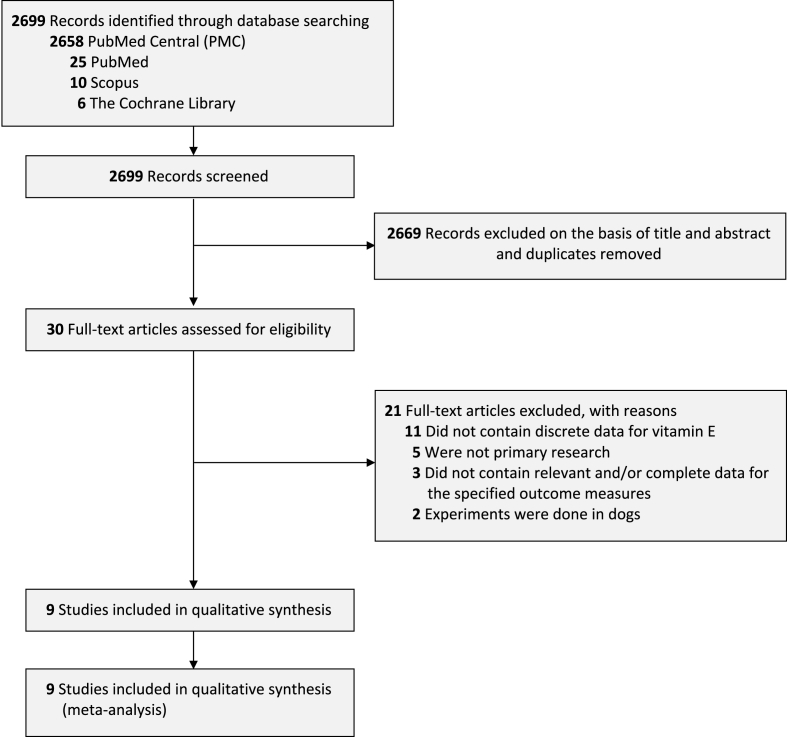
PRISMA diagram of the detailed process of selection of studies for inclusion in the systematic review and meta-analysis.

Initial searching from the database found 2,699 studies, from which 30 studies were found eligible after titles and abstracts screening as well as removing the duplicates. Of these eligible studies, 20 articles were further excluded after the full-text screening. Eleven articles did not contain discrete data for vitamin E, five articles were not primary research, three articles did not provide the relevant data about the specified outcome of interest, and two articles were done in dogs thus resulting in the final number of 9 studies [[10], [11], [12], [13], [14], [15], [16], [17], [18]] for the analysis (Fig. 1). Out of 9 studies, five studies use Wistar rats with weights ranging from 180 to 570 g, three studies use Sprague-Dawley rats weighing from 200 to 220 g, and the remaining one study uses Swiss white mice. Most of the included studies use the adhesion induction technique described by Hemadeh O et al. [12] in which gauze was utilized to rub the wall of the abdominal organs until the hemorrhagic points appeared and one drop of alcohol was instilled into those walls. Regarding the vitamin E intervention, almost all of the included studies use 10 mg of vitamin E dissolved in 5 ml olive oil and injected intraperitoneally as the way of administration. The details of the characteristics of each included study are summarized in Table 1.

**Table 1:** Characteristics of included studies.

Study	Sample characteristics	Adhesion induction procedure	Vitamin E dosage	Adhesions Results	
				Vitamin E groups	Control groups
Corrales F et al. [9] 2008	Wistar male rats, weighing 450–550 g	(1) Anesthesia (2) Trichotomy (3) Abdominal incision 4 cm (4) Exposing wall of cecum (5) Rubbing cecum with gauze until hemorrhagic points appeared (6) One drop of absolute alcohol applied to the cecum wall (7) Cecum returned to the abdominal cavity (8) Parietal peritoneum clamped with hemostat and 3-0 silk suture ligature was placed (9) Abdominal cavity closed with silk 3-0	n = 15 10 mg vitamin E in 5 ml olive oil, injected intraperitoneally	- Grade 0 = 8 - Grade 1 = 3 - Grade 2 = 2 - Grade 3 = 1 - Grade 4 = 1	- Grade 0 = 0 - Grade 1 = 0 - Grade 2 = 5 - Grade 3 = 7 - Grade 4 = 3
de la Portilla F et al. [10] 2004	Wistar Albino rats, weighing 380–570 g	(1) Anesthesia with 50 mg/kg ketamine (2) Laparotomy via 4 cm midline incision (3) Exposing the cecum (4) Rubbing cecum with gauze until hemorrhagic points appeared (5) One drop of absolute alcohol applied to the cecum wall (6) Cecum returned to the abdominal cavity (7) Parietal peritoneum clamped with hemostat and 3-0 silk suture ligature was placed (8) Abdominal cavity closed with silk 3-0	n = 14 10 mg vitamin E in 5 ml olive oil, injected intraperitoneally	- Grade 0 = 3 - Grade 1 = 8 - Grade 2 = 3 - Grade 3 = 0 - Grade 4 = 0	- Grade 0 = 0 - Grade 1 = 0 - Grade 2 = 1 - Grade 3 = 8 - Grade 4 = 5
Durmus AS et al. [11] 2011	Sprague-Dawley rats, weighing 200–220 g	(1) Anesthesia with 85 mg/kg ketamine and 6 mg/kg xylazine (2) Abdomens shaved and prepared with 1% povidone iodine (3) Laparotomy via 3 cm midline incision (4) Small bowel was retracted and uterus exposed (5) Scrapping the abdominal sidewall and antimesenteric surface of left uterine horn with no. 15 scalpel blade, creating punctate serosal hemorrhages (6) Abdominal incision was closed	n = 10 10 mg vitamin E in 2 ml 0.9% saline solution, injected intraperitoneally	Mean adhesion scores: 2.6 ± 0.15	Mean adhesion score: 2.9 ± 0.1
Hemadeh O et al. [12] 1993	Sprague-Dawley rats	(1) Anesthesia (2) Abdominal incision 4 cm, exposing wall of cecum (3) Rubbing cecum with gauze until hemorrhagic points appeared (4) One drop of absolute alcohol applied to the cecum wall (5) Cecum returned to the abdominal cavity (6) Parietal peritoneum clamped with hemostat and 3-0 silk suture ligature was placed (7) Abdominal cavity closed with silk 3-0	n = 15 30 IU/kg body weight of vitamin E	- No adhesions: 5 - Significant adhesions: 10	- No adhesions: 0 - Significant adhesions: 15
Kagoma P et al. [13] 1985	Adult female Swiss white mice, weighing 20–25 g	(1) Anesthesia with ether (2) Laparotomy through 2.5 cm midline incision (3) Right parietal peritoneum firstly pinched with fine hemostat (4) Pinched peritoneal fold was then ligated with 3–0 silk suture (5) Abdominal incision closed with a continuous 3–0 nylon suture	n = 62 300 IU/kg body weight of vitamin E in 20 ml of 100% ethyl alcohol, added into the chow	- Adhesions: 36 - Mean grade of adhesions: 1.1 ± 0.1	- Adhesions: 60 - Mean grade of adhesions: 2.4 ± 0.18
Putra RAN et al. [14] 2017	Wistar white rats, weighing 200–300 g	(1) Anesthesia with ketamine 0.36 mg and diazepam 0.3 mg (2) Laparotomy through midline incision 3 cm (3) Intestinal resection 1 cm with 15 cm distance from ileocecal junction → ileal abration (4) Intraperitoneal wound was closed with single-layer end-to-end technique with 3–0 silk	n = 6 10 mg vitamin E in 5 ml soybean oil, given topically into intraperitoneal	- Grade 0 = 0 - Grade 1 = 3 - Grade 2 = 2 - Grade 3 = 1 - Grade 4 = 0 - Mean = 1.66 ± 0.81	- Grade 0 = 0 - Grade 1 = 0 - Grade 2 = 0 - Grade 3 = 2 - Grade 4 = 4 - Mean = 3.66 ± 0.51
Yetkin G et al. [15] 2009	Wistar-Albino rats, weighing 180–220 g	(1) Anesthesia with 50 mg/kg ketamine (2) Laparotomy via 4 cm midline incision (3) Exposing the cecum (4) Rubbing cecum with gauze until hemorrhagic points appeared (5) One drop of absolute alcohol applied to the cecum wall (6) Cecum returned to the abdominal cavity (7) Parietal peritoneum clamped with hemostat and 3-0 silk suture ligature was placed (8) Abdominal cavity closed with silk 3-0	n = 14 10 mg vitamin E in 5 ml olive oil, injected intraperitoneally	- Grade 0 = 0 - Grade 1 = 3 - Grade 2 = 8 - Grade 3 = 3 - Grade 4 = 0 - Mean = 1 ± 0.67	- Grade 0 = 0 - Grade 1 = 0 - Grade 2 = 1 - Grade 3 = 8 - Grade 4 = 5 - Mean = 3.28 ± 0.61
Yildiz H et al. [16] 2011	Sprague-Dawley, weighing 200–220 g	(1) Anesthesia with 85 mg/kg ketamine and 6 mg/kg xylazine (2) Abdominal skin was shaved and antisepsis with 10% povidone iodine (3) Laparotomy via 3 cm midline incision (4) Small bowel retracted and uterus was exposed (5) Scrapping the abdominal sidewall and antimesenteric surface of left uterine horn with no. 15 scalpel blade, creating punctate serosal hemorrhages (6) Abdominal incision was closed with 4–0 silk sutures	n = 10 2 ml (300 mg) vitamin E dissolved in 58 ml olive oil, injected intraperitoneally	Mean adhesion score: 2 ± 0.2	Mean adhesion score: 2.7 ± 0.15
Yulianto W et al. [17] 2017	Wistar white rats, weighing 200–300 g	(1) Anesthesia with ketamine 0.36 mg and diazepam 0.3 mg (2) Laparotomy through midline incision 3 cm (3) Intestinal resection 1 cm with 15 cm distance from ileocecal junction → ileal abration (4) Intraperitoneal wound was closed with single-layer end-to-end technique with 3–0 silk	n = 6 10 mg vitamin E in 5 ml soybean oil, given topically into intraperitoneal	- Grade 0 = 0 - Grade 1 = 3 - Grade 2 = 2 - Grade 3 = 1 - Grade 4 = 0 - Mean = 1.66 ± 0.81	- Grade 0 = 0 - Grade 1 = 0 - Grade 2 = 0 - Grade 3 = 2 - Grade 4 = 4 Mean = 3.66 ± 0.51

### 3.2 Quality of study assessment

SYRCLE's RoB tool was used to evaluate the quality assessment of animal experimental studies. A breakdown of the individual categories of SYRCLE's RoB tool led to the following results: 100% of the included studies reported sequence generation and baseline characteristics; none of the studies reported allocation concealment; 44.4% reported random housing; none of the studies reported performance blinding; 100% reported random outcome assessment; 22.2% reported detection blinding; 55.5% reported incomplete outcome data; 100% showed a low risk of selective outcome reporting, and 44.4% showed a low risk of bias regarding other sources of bias. The detailed results of SYRCLE's RoB tool for each of the included studies are summarized in Table 2.

**Table 2:** Risk of bias assessment using RoB v2.

Study	Selection bias			Performance bias		Detection bias		Attrition bias	Reporting bias	Other
	Sequence generation	Baseline characteristics	Allocation concealment	Random housing	Blinding	Random outcome assessment	Blinding	Incomplete outcome data	Selective outcome reporting	Other sources of bias
Corrales F et al. [9] 2008	+	+	–	–	?	+	+	+	+	+
de la Portilla F et al. [10] 2004	+	+	–	–	?	+	+	+	+	+
Durmus AS et al. [11] 2011	+	+	–	+	–	+	–	–	+	–
Hemadeh O et al. [12] 1993	+	+	–	–	–	+	–	–	+	–
Kagoma P et al. [13] 1985	+	+	–	+	–	+	–	–	+	–
Putra RAN et al. [14] 2017	+	+	–	+	–	+	–	+	+	+
Yetkin G et al. [15] 2009	+	+	–	–	–	+	?	+	+	?
Yildiz H et al. [16] 2011	+	+	–	–	–	+	–	–	+	–
Yulianto W et al. [17] 2017	+	+	–	+	–	+	–	+	+	+

(+) indicates low risk of bias, (−) indicates high risk of bias, (?) indicates unclear risk of bias.

### 3.3 Vitamin E and substantial peritoneal/intra-abdominal adhesions

**Fig. 2:**
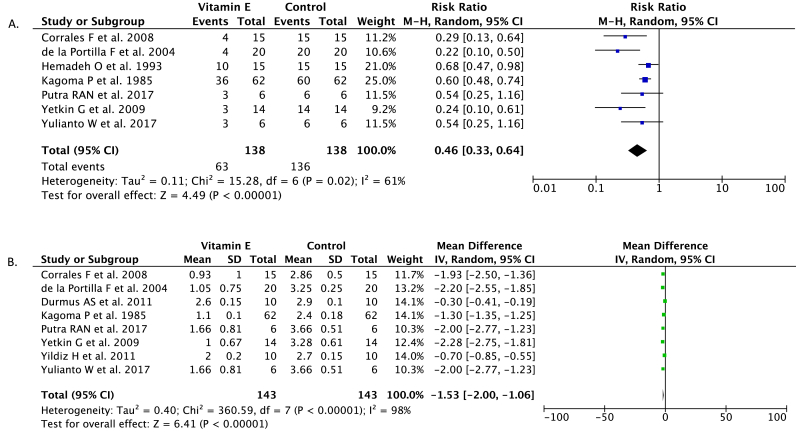
Forest plot that demonstrates the association between vitamin E administration with substantial peritoneal/intra-abdominal adhesions (A) and mean grade of adhesions outcomes (B).

Seven studies (n = 276) reported the incidence of substantial peritoneal/intra-abdominal adhesions as the outcome of vitamin E administration compared with control in rats models. Our pooled analysis revealed that vitamin E administration may reduce the risk of substantial peritoneal/intra-abdominal adhesions in rats models (RR 0.46; 95%CI: 0.33–0.64, *p* < 0.00001, random-effect modeling) (Fig. 2A). Heterogeneity was statistically significant with I^2^ = 61%, p = 0.02.

### 3.4 Vitamin E and mean grade of peritoneal/intra-abdominal adhesions

The mean grade of peritoneal/intra-abdominal adhesions outcome was revealed in eight studies (n = 286). The pooled estimate indicated that vitamin E administration was associated with a lower mean grade of peritoneal/intra-abdominal adhesions (Mean Difference −1.53; 95%CI: −2.00, −1.06, *p* < 0.00001, random-effect modeling) (Fig. 2B). Heterogeneity was statistically significant with I^2^ = 98%, p < 0.00001.

### 3.5 Publication bias

In each of the outcomes of interest in this study, the number of included studies was less than 10 studies. In this case, the funnel plots and statistical tests for publication bias detection are not reliable when compared with whenever there are more than 10 included studies in each outcome [19,20]. Therefore, publication bias analysis was not performed in this study.

## 4 Discussion

According to our pooled analysis, it was discovered that vitamin E administration was associated with a reduction of peritoneal/intra-abdominal adhesions and mean grade of adhesions in rat models.

There are some explanations of how vitamin E administration could reduce the incidence of peritoneal/intra-peritoneal adhesions. First, several studies have shown that reactive oxygen species (ROS) may play a role in adhesion formation following surgical procedures. Those studies showed that after surgery, the levels of free radicals, such as superoxide anions, xanthine oxidase, and malondialdehyde (MDA) increased significantly [21,22]. The increase in the levels of those molecules may result in the lipid peroxidation process which can damage proteins, lipids, and cells within the body [23]. ROS may also contribute to a series of cascades which includes a reduction in the bioavailability of nitric oxide that will cause oxidative damage and in turn result in dysfunction and remodeling of the vascular wall, impairing the endothelial cell growth and endothelium-dependent vasodilatation, causing apoptosis, stimulating the migration of endothelial cells, and activation of adhesion molecules [24]. All of these processes are involved in the pathogenesis of adhesion formation. Vitamin E can serve as a natural antioxidant that prevents the accumulation of peroxides and protects the cells from free radical damage [25,26]. Vitamin E may also reduce the effects of oxidative stress in living cells by acting as a scavenger for locally created free radicals. Vitamin E provides a cellular defense mechanism that prevents peroxidative processes by sequestering free radicals, CAT, and GPx, which detoxifies peroxides and protects cells from subsequent deleterious effects, therefore may prevent the adhesion formation process [27]. Second, inflammation caused by intra-abdominal trauma can also be one of the culprits for adhesion formation. Vitamin E can serve as an anti-inflammatory agent by inhibiting cyclooxygenase-2 (COX-2) [28,29] and endogenous conversion of arachidonic acid into PGE_2_ and PGF_2α_ [30,31] therefore reducing the inflammatory process triggered by peritoneal trauma. Vitamin E also inhibits several pro-inflammatory cytokines, especially IL-1 and IL-6 which are responsible for adhesion formation [32,33]. Anti-inflammatory properties of vitamin E also cover up its ability to inhibit platelet adhesion and release, which is known to represent one of the early events in lesion repair [34,35]. By diminishing the platelet aggregation process, the amount of fibroblast and smooth-muscle mitogens released by activated platelet will be decreased; moreover, thromboplastin and resultant fibrin generation may also be reduced, therefore reducing the formation of adhesions [36]. Finally, vitamin E has an anti-fibroblastic effect in which it will inhibit the proliferation of various cell lines, including fibroblasts [[37], [38], [39]]. To be noted, the fibroblast is one of the cells which is largely responsible for adhesion formation. Moreover, vitamin E also inhibits TGF-β, a strong inducer of fibrosis, and reduces collagen production, apparently because of inhibition of the expression of collagen α_1_ and protein kinase C (PKC) [40].

This study still has several limitations. We only found a relatively small number of studies for the analysis because there are currently a limited number of studies that specifically assess the benefit of vitamin E in adhesion prevention. Most of the included studies also only have a small number of samples and showed a relatively high risk of bias according to SYRCLE's RoB tool, especially on the performance and detection blinding, also on the allocation concealment process. Significant heterogeneities were also found in all outcomes of interests included in this study. This might be caused by differences in the species of rats used as samples and in the vitamin E dosages used in each of the included studies. More studies with larger sample sizes and good methodological qualities which focus on the prevention of peritoneal/intra-abdominal adhesion through administration of vitamin E in animal models are still needed to confirm the results of our study. Further studies in humans, probably in the form of case series or cohorts first, might also consider trying vitamin E as one of the supplemental agents for patients undergoing abdominal surgeries to prevent the occurrence of adhesions.

## 5 Conclusion

Our systematic review and meta-analysis indicated that vitamin E administration in rats models was associated with a risk reduction of substantial peritoneal/intra-abdominal adhesions and mean grade of adhesions. This review proposes that vitamin E supplementation might give benefit to prevent peritoneal/intra-abdominal adhesions in those who undergo abdominal surgeries. Even so, more in-vivo studies with larger sample sizes and proper methods and Covid-19 are still required to further verify the results from our study. If possible, studies in human samples about vitamin E benefit in the prevention of peritoneal/intra-abdominal adhesions might also be needed.

## Provenance and peer review

Not commissioned, externally peer-reviewed.

## Ethics approval and consent to participate

Not applicable.

## Consent for publication

Not applicable.

## Availability of data and materials

Data analyzed in this study were a re-analysis of existing data, which are openly available at locations cited in the reference section.

## Funding

None.

## Authors’ contributions

TS: conceptualization, methodology, formal analysis, data curation, writing‐original draft, visualization, writing‐review, and editing. MH: conceptualization, methodology, formal analysis, data curation, writing‐original draft, writing‐review and editing. PP: conceptualization, methodology, formal analysis, data curation, writing‐original draft, writing‐review and editing. AB: conceptualization, validation, supervision, writing‐review and editing. TRT: conceptualization, validation, supervision, writing‐review and editing. HL: conceptualization, validation, supervision, writing‐review and editing. All authors read and approved the final manuscript.

## Sources of funding

There is no sponsor for this study, all funding from my self.

## Guarantor

Prof. Mochammad Hatta.

## Declaration of competing interest

The authors declare that they have no competing interests.
