# Supplementary material for: Vitamin E administration as preventive measures for peritoneal/intra-abdominal adhesions: A systematic review and meta-analysis
Source: Ann Med Surg (Lond). 2022 Jul 31;80:104225. doi: 10.1016/j.amsu.2022.104225 (PMC9422189; doi:10.1016/j.amsu.2022.104225)
Supplement: Multimedia component 1 [file mmc1.docx]

**Supplementary Table I. Literature search strategy**

**1A. PubMed Search String:**

“(peritoneal OR peritoneum OR abdominal OR abdomen OR bowel) AND (adhesion OR fibrosis OR scarring) AND (vitamin E OR alpha-tocopherol)”

**1B. Example Scopus Search Strategy:**

1. peritoneal.mp.

2. peritoneum.mp.

3. abdominal.mp.

4. abdomen.mp.

5. bowel.mp.

6. adhesion.mp.

7. fibrosis.mp.

8. scarring.mp.

9. vitamin E.mp.

10. alpha-tocopherol.mp.

11. 1 or 2 or 3 or 4 or 5

12. 6 or 7 or 8

13. 9 or 10

14. 11 and 12 and 13
